# Environment and systemic autoimmune rheumatic diseases: an overview and future directions

**DOI:** 10.3389/fimmu.2024.1456145

**Published:** 2024-09-10

**Authors:** May Y. Choi, Karen H. Costenbader, Marvin J. Fritzler

**Affiliations:** ^1^ Department of Medicine, Cumming School of Medicine, University of Calgary, Calgary, AB, Canada; ^2^ McCaig Institute for Bone and Joint Health, Calgary, AB, Canada; ^3^ Department of Medicine, Div of Rheumatology, Inflammation and Immunity, Brigham and Women’s Hospital, Boston, MA, United States; ^4^ Medicine, Harvard Medical School, Boston, MA, United States

**Keywords:** autoimmunity, autoimmune diseases, environment, autoantibodies, epigenetics, microbiome, machine learning, artificial intelligence

## Abstract

**Introduction:**

Despite progress in our understanding of disease pathogenesis for systemic autoimmune rheumatic diseases (SARD), these diseases are still associated with high morbidity, disability, and mortality. Much of the strongest evidence to date implicating environmental factors in the development of autoimmunity has been based on well-established, large, longitudinal prospective cohort studies.

**Methods:**

Herein, we review the current state of knowledge on known environmental factors associated with the development of SARD and potential areas for future research.

**Results:**

The risk attributable to any particular environmental factor ranges from 10-200%, but exposures are likely synergistic in altering the immune system in a complex interplay of epigenetics, hormonal factors, and the microbiome leading to systemic inflammation and eventual organ damage. To reduce or forestall the progression of autoimmunity, a better understanding of disease pathogenesis is still needed.

**Conclusion:**

Owing to the complexity and multifactorial nature of autoimmune disease, machine learning, a type of artificial intelligence, is increasingly utilized as an approach to analyzing large datasets. Future studies that identify patients who are at high risk of developing autoimmune diseases for prevention trials are needed.

## Introduction

Environmental factors operating on the background of hormonal factors and genetic vulnerability may be accelerating factors included in a long-held paradigm that helps explain the etiology of systemic autoimmune rheumatic disease (SARD), including systemic lupus erythematosus (SLE), rheumatoid arthritis (RA), systemic sclerosis (SSc), Sjögren’s disease (SjD), idiopathic inflammatory myopathies (IIM) and others (1). On the backdrop of an increasing prevalence of SARD and other autoimmune diseases (2–6), potential accelerating factors include several environmental and socioeconomic factors that include alterations of foods, increasing exposure to xenobiotics due to water and air pollution, heat and other extreme weather events (i.e., climate change), biodiversity loss, ultraviolet (UV) light exposure, pandemics and infections, and socioeconomic factors such as changes in personal lifestyles and psychological stress.

Extensive research over the past three to four decades has elucidated the environmental factors associated with SLE (7) and other SARD. In general, the environmental factors can be classified as airborne, waterborne, workplace/occupational, social, and behavioral (8). While it has not been possible to identify a universal environmental “pathogen” for all SARD, there is compelling evidence that some environmental exposures clearly serve as risk factors for disease onset. The central importance of identifying these factors is that many of these factors are actionable and modifiable through intervention and remediation. Expanding the use of machine learning (ML), a form of artificial intelligence (AI), to analyze large datasets including environmental exposures may lead to the identification of other modifiable environmental risk factors, and allow the development of new disease-specific remediation programs (2).

## Environmental factors and autoimmunity

The development of SARD has been associated with several lifestyle behaviors. For instance, cigarette smoke (9–11), obesity (12), alcohol use (moderate consumption being protective) (10, 13–15), poor nutrition and intake of ultra-processed foods (16), psychosocial factors (e.g., major depression (17), sleep deprivation (18), child abuse, personal trauma, post-traumatic stress disorder [PTSD]) (19, 20), and reproductive factors (21–23) have been associated with SLE development. Environmental exposures such as air pollution (24), occupational hazards (25), residential proximity to hazardous waste sites or pesticide exposure (26, 27), UV light (28–33), vitamin D deficiency (34), and exposure to viruses (35, 36) have also been linked to increased SLE risk. Similar lifestyle factors have been reported for increased risk of developing RA (moderate alcohol consumption decreases RA risk), SSc, IIM, other SARD, and autoinflammatory conditions (**Tables 1**, **2**).

**Table 1:** Environmental factors that increase risk for systemic autoimmune rheumatic diseases.

Lifestyle Exposure	Disease Association	Reported Risk from Select Key References (Citation)
Air Pollution	RA	• HR 1.31 (95%CI: 0.98–1.74) living near traffic pollution (road) vs. not (24)
	SLE	• Increases in air pollutants nitrogen dioxide (NO_2_), carbon monoxide (CO), and fine particles (PM_2.5_) (HR 1.21 [95% CI: 1.08–1.36], HR 1.44 [95% CI: 1.31–1.59], and HR 1.12 [95% CI: 1.02–1.23], respectively) (37)
	SARD^1^	• OR 1.13 (95%CI: 1.02-1.25) for lowest vs. highest satellite fine particulate air pollution level (38)
Cigarette Smoke	RA	• RR 3.8 (95%CI: 2.0-6.9) in current smokers vs. never smokers (39) • OR 1.65 (95%CI: 1.03–2.64) for >20 versus 0 pack-years) for anti-CCP-positive RA (40)
	SLE	• OR 1.50 (95%CI: 1.09–2.08) for current smokers compared with non-smokers (11) • HR 1.86 (95%CI: 1.14–3.04) for current vs. never smokers for dsDNA+ SLE risk (9)
Diet	SLE	• Women in the highest tertile of cumulatively updated dietary ultra-processed food (UPF) intake/day were at almost 50% greater risk of developing SLE vs. women in the lowest tertile of UPF daily intake (16)
Hazardous Waste Sites	SLE	• Exposure to volatile organic compounds (P < 0.05) (26)
Obesity	RA	• History of obesity (OR 1.24 [95%CI: 1.01–1.53]) (41)
	SLE	• An 85% (HR 1.85 [95%CI: 1.17-2.91]) significantly increased risk of SLE among obese compared to normal BMI women in the more recent NHSII cohort (12), but not NHS
Organic Solvents, Pesticides and Heavy Metal	RA	• Application of chemical fertilizers (adjusted OR 1.7 [95%CI: 1.1-2.7]) and cleaning with solvents (OR 1.6 [95%CI: 1.1-2.4]) (42)
	SLE	• Pesticide exposure (adjusted OR 2.24 [95%CI: 1.28–3.93]) (27) • Association with SLE risk seen with mercury (OR 3.6 [95%CI: 1.3-10.0]) and mixing pesticides for agricultural work (OR 7.4 [95%CI: 1.4-40.0]) (43)
	SSc	• OR 2.9 (95%CI: 1.1-7.6) for solvent organic solvent exposure (male SSc vs controls) (44)
Periodontitis	RA	• OR 1.16 (95%CI: 1.13-1.21) history of periodontitis (45)
Psychosocial	SLE	• Probable PTSD (HR 2.94 [95%CI: 1.19–7.26]) and trauma exposure (HR 2.83 [95%CI: 1.29–6.21]) (19) • Women with a history of depression vs. no depression (HR 2.67 [95%CI: 1.91-3.75]) (17) • Adverse childhood experiences (abuse, neglect, and household challenges) associated with increased risk of SLE. Exposure to the highest vs. lowest physical and emotional abuse was associated with 2.57 times greater risk of SLE (95%CI: 1.30–5.12) (46). HR for ≥2 episodes of severe sexual abuse compared to no abuse was 2.51 (95%CI: 1.29–4.85) and ≥5 episodes of severe physical abuse was 2.37 (95%CI: 1.13–4.99) among Black women) (20).
Reproductive/ Hormonal Factors	SLE	• Pooled RR 1.5 (95%CI: 1.1-2.1) oral contraceptive use and use of postmenopausal hormones RR 1.9 (95%CI: 1.2-3.1) (21)
Silica	RA	• Silica exposed men OR 2.2 (95%CI: 1.2-3.9) among men aged 18 to 70 years and 2.7 (95%CI: 1.2-5.8) among those aged 50 to 70 years (47)
	SLE	• Medium silica exposure was OR 2.1 (95%CI: 1.1–4.0), high exposure OR 4.6 (95%CI: 1.4–15.4) (25)
	Vasculitis	• Overall significant summary effect estimate of silica “ever exposure” with development of AAV (OR 2.56 (95%CI: 1.51–4.36) (48)
	SSc	• The combined estimator of relative risk for studies in females was 1.03 (95%CI: 0.74–1.44) and was 3.02 (95%CI: 1.24–7.35) for males (49).
Sleep Deprivation	SLE	• HR 2.47 (95%CI: 1.29-4.75) for chronic low sleep duration (≤5 hours/night versus >7–8 hours) (18)
UV Radiation	SLE	• History of more than one serious sunburn before the age of 20 years (OR 2.2, 95%CI: 1.2–4.1) and sunburn-susceptible skin type (OR 2.9, 95%CI: 1.6–5.1) (32)
Viruses	SLE	• Epstein-Barr virus serologic reactivation among unaffected SLE relatives (viral capsid antigen IgG OR 1.28 [95%CI: 1.07-1.53], p=0.007 and early antigen IgG OR 1.43 [95%CI: 1.06-1.93], p=0.02) (36)
	SARD	• Higher risk of RA (adjusted HR (aHR) 2.98 [95%CI: 2.78–3.20]), SLE (aHR 2.99 [95%CI: 2.68–3.34]), dermatopolymyositis (aHR 1.96 [95%CI: 1.47–2.61]), SSc (aHR 2.58 [95%CI: 2.02–3.28]), SjD (aHR 2.62 [95%CI: 2.29–3.00]), mixed connective tissue disease (aHR 3.14 [95%CI: 2.26–4.36]), Behçet's disease (aHR 2.32 [95%CI: 1.38–3.89]), polymyalgia rheumatica (aHR 2.90 [95%CI: 2.36–3.57]), and vasculitis (aHR 1.96 [95%CI: 1.74–2.20]) among COVID-19 vs. non-COVID-19 exposed unvaccinated individuals (50).

AAV, anti-neutrophil cytoplasmic autoantibody (ANCA)-associated vasculitis; CI, confidence interval; CCP, cyclic citrullinated peptide; HR, hazard ratio; NHSII, Nurses’ Health Study Cohort 2; OR, odds ratio; RA, rheumatoid arthritis; RR, relative risk; SARD, systemic autoimmune rheumatic diseases; SjD, Sjögren disease; SLE, systemic lupus erythematosus; SSc, systemic sclerosis; UV, ultraviolet.

1. SARD included systemic lupus erythematosus, Sjögren's disease, scleroderma, polymyositis, dermatomyositis, or undifferentiated connective tissue disease.

**Table 2:** Environmental factors that decrease risk for systemic autoimmune rheumatic diseases.

Lifestyle Exposure	Disease Association	Reported Risk from Select Key References (Citation)
Alcohol	RA	• HR 0.78 (95%CI: 0.61–1.00) for alcohol use of 5.0–9.9 gm/day (51)
	SLE	• HR 0.65 [95%CI: 0.45–0.96] among women who drank 2 or more servings of wine had significantly decreased SLE risk compared to women who did not drink wine (13)
Diet	RA	• HR 0.67 (95%CI: 0.51-0.88) among women aged ≤55 years, better quality diet was associated with lower RA risk, particularly seropositive RA (52)
Exercise	SLE	• Regular exercise (performing at least 19 metabolic equivalent hours of exercise per week) assessed with other healthy behaviors (never or past smoker, healthy diet, moderate alcohol consumption, healthy body weight) was associated with a 19% reduction in SLE risk per additional healthy behavior, such that women with four or more healthy lifestyle factors had the lowest risk (HR 0.42 [95%CI: 0.25-0.70]) (53).
	RA	• Similar to the SLE study above, a lower risk of RA was also observed with a healthier lifestyle including regular exercise, i.e., women with five healthy lifestyle factors had the lowest risk (HR 0.42 [95%CI: 0.22-0.80]) (54).
Reproductive/ Hormonal Factors	RA	• RR 0.8 (95%CI: 0.6–1.0) for breastfeeding for 2–23 total months (55)
Vitamin D	SARD^1^	• Vitamin D 2000IU daily supplementation was associated with a 22% reduction in the development of autoimmune disease (HR 0.78 [95% CI: 0.61, 0.99], P=0.05) (56).

CI, confidence interval; HR, hazard ratio; RA, rheumatoid arthritis; RR, relative risk; SARD, systemic autoimmune rheumatic diseases; SLE, systemic lupus erythematosus; 1. This included RA, polymyalgia rheumatic, autoimmune thyroid disease, psoriasis, inflammatory bowel disease, and many others (e.g., SLE, systemic sclerosis).

**Figure 1:**
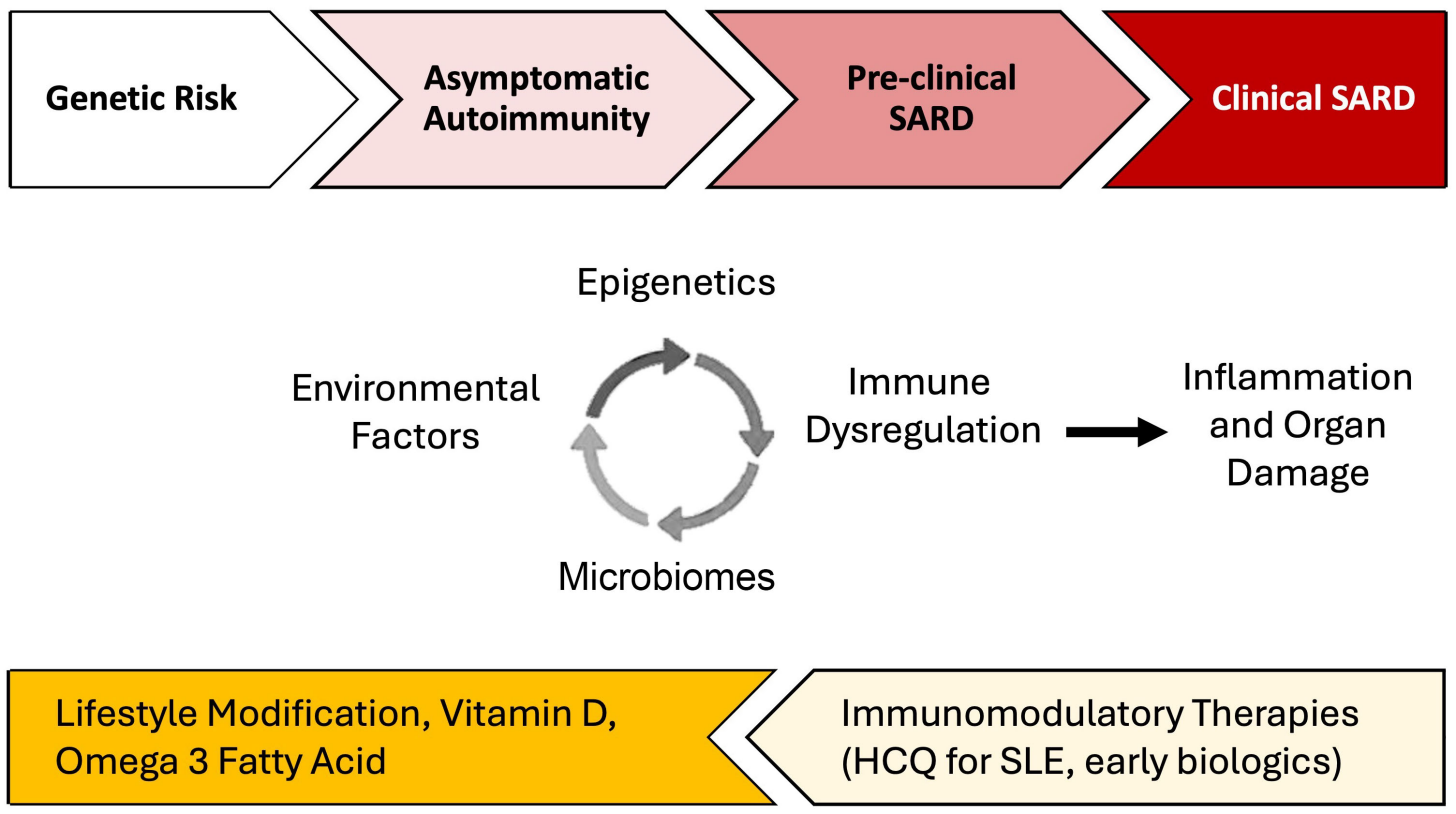
Environmental factor-associated pathogenesis and personalized preventative vs. treatment interventions for systemic autoimmune rheumatic diseases (SARD). Among individuals genetically predisposed to SARD development, unhealthy lifestyle behaviors and other environmental factors can trigger dysregulation in the microbiome, epigenetic changes, and immune dysregulation which, together, drive inflammation. In turn, inflammation can drive further derangements in the microbiome, cause distinct epigenetic changes, and lead to additional immune dysregulation. During the periods of asymptomatic autoimmunity and pre-clinical SARD, this positive feedback leads to a process wherein inflammation becomes chronic and self-sustaining, ultimately driving autoimmunity and eventually leading to organ damage and clinical disease. Effective lifestyle interventions, supplementation, and early introduction of immunomodulatory therapies may help prevent disease progression. There may be a potential role for treatments such as hydroxychloroquine for pre-SLE [SMILE trial underway (61)] and Abatacept, a T-cell co-stimulation inhibitor, for pre-RA (62, 63).

Precisely how and the extent to which these lifestyle factors contribute to individual risk of autoimmune disease likely varies (57, 58). This has been particularly well-studied using large cohort studies including cohorts enrolled in the Nurses’ Health Study (NHS) and Black Women’s Health Study (BWHS). In SLE, each factor independently increases the risk of disease development by 10-200%, but they likely interact with each other and with genetic risk, potentially synergistically, to accelerate brewing autoimmunity in SLE [reviewed in (57–60)]. Using SLE as an example below, we discuss several potential biologic pathways involving epigenomics, the microbiome, and immune dysregulation that lead to inflammation and organ damage, mechanisms that may also apply to the development of other SARD (**Figure 1**).

## Common pathways of pathogenesis: immune dysregulation, epigenomics, the microbiome

### Immune dysregulation

Inflammation is an adaptive response to stressors that involves multiple physiological processes that include the innate and adaptive immune systems. In turn, inflammation regulates – and is regulated by – several highly interconnected systems including the epigenome and microbiome (64). Unhealthy lifestyle behaviors (i.e., smoking, sedentary lifestyle, and consumption of ultra-processed foods) promote systemic inflammation leading to chronic inflammatory diseases, including SARD. Before developing overt clinical manifestations, individuals developing SARD have a period of asymptomatic autoimmunity and inflammation of variable intensity and duration, characterized by increasing oxidative stress, loss of immune tolerance, autoantibody formation, immune complex deposition and complement activation, epigenetic modifications, and upregulation and/or downregulation of cytokine expression [reviewed in (65)].

In SLE, both obesity and exposure to the toxic components of cigarette smoke induce oxidative stress (66). This, in turn, raises intracellular levels of reactive oxygen species that damage DNA producing immunogenic DNA adducts that can lead to the production of ‘pathogenic’ anti-double-stranded DNA antibodies (dsDNA) (67–69). In the NHS and NHSII cohorts, smokers were at higher risk of developing anti-dsDNA positive SLE compared to never-smokers (hazard ratio [HR] 1.86 [95% confidence interval (CI): 1.14-13.04]), while there were no significant associations between smoking status or pack-years and overall SLE or anti-dsDNA negative SLE (9). In addition to elevated oxidative stress, the byproducts of smoking could also augment native autoreactive B cells (11) and induce pulmonary antinuclear antibody (ANA) as demonstrated in the lungs of exposed mice (70). Smoking may also influence specific genes in the pathogenesis of SLE (57). An individual with a high SLE genetic risk score or GRS (score based on 86 single-nucleotide polymorphisms and 10 classic HLA alleles previously associated with SLE) and a status of current/recent smoking was strongly associated with SLE risk (odds ratio [OR] 1.5, p=0.0003 versus more distant past/never smoking) and even stronger in the presence of anti-dsDNA antibodies. Not surprisingly, smoking also affects circulating cytokines. Elevated SARD-related cytokines including the B-cell lymphocyte stimulator (BlyS) (70), tumor necrosis factor-alpha (TNF-α), and interleukin (IL)-6 (71, 72), but lower IL-10 (an anti-inflammatory cytokine) have been detected in smokers (73). These cytokines affect the function of T cells and CD4^+^ regulatory T cells, which are important in maintaining self-tolerance. Similarly, adipose tissue, in particular visceral fat, secretes pro-inflammatory adipocyte-derived cytokines and exhibits higher levels of C-reactive protein (CRP), TNF-α receptor 2, and IL-6 than non-obese individuals (74).

The association between SLE risk and diet is less clear in humans (75–77) compared to other autoimmune diseases such as RA [reviewed in (78)]. There is evidence from SLE-prone mice models that low dietary fiber intake and a Western-type diet (i.e., high in sugar, fat, refined grains, and red meat) are associated with increased autoantibody production (79, 80). In the BWHS, a diet high in carbohydrates and low in fats was associated with an increased risk of developing SLE in African American women (HR 1.88 [95%CI: 1.06-3.35]) (75). Consumption of ultra-processed foods, in particular sugar and artificially sweetened beverages, has been associated with an increased risk of developing SLE among women (16). Low to moderate alcohol consumption (approximately 1/2 drink a day), on the other hand, has been shown to reduce the risk of SLE development among women (10, 13–15). Alcohol (e.g., ethanol) and antioxidants may counteract the changes induced by smoking and obesity, i.e., inhibiting key enzymes in DNA synthesis and suppressing TNF-α, IL-6, IL-8, and interferon (IFN)-γ that lower systemic inflammation (81, 82).

Several studies have reported an association between lack of sleep and SLE risk in humans (18, 83, 84). In the NHS and NHSII cohorts, chronic low sleep duration (</=5 hours/night versus the recommended >7-8 hours) was associated with increased SLE risk (adjusted HR 2.47 [95% CI: 1.29, 4.75]), with stronger effects among those with body pain and depression. In sleep-deprived individuals, increased levels of IL-6 and TNF-α have been reported (85–89). In SLE-prone mice, sleep deprivation was associated with accelerated production of autoantibodies and earlier disease onset (90). Sleep disturbances arising in individuals who have had childhood or adult trauma, PTSD, or occupational stress from working night or rotating shifts, may also explain why these factors have also been linked to SLE onset (17, 19, 20, 43, 91, 92). In the NHSII, PTSD, a condition arising after exposure to trauma and marked by severe psychological stress, was associated with increased SLE risk (HR 2.94 [95% CI: 1.19-7.26], p<0.05) compared to women with no trauma, even after adjusting for other SLE risk factors smoking, body mass index (BMI), and oral contraceptive use (19). In the NHS and NHSII, women with a history of depression had a higher risk of SLE (HR 2.67 [95:CI: 1.91-3.75] p<0.001) compared to women with no depression (17). Systemic inflammation, denoted by elevated TNF, IL-6, and CRP levels, has been repeatedly reported in individuals with emotional stress and distress (91, 93–102).

There is also evidence that sex hormones are important in SLE development (21, 22), a disease, like some other SARD, that predominantly affects females. In SLE, a population-based nested case-control study using the UK’s General Practice Research Database demonstrated that there was a dose-response in oral contraceptive pill (ethinyl estradiol) and SLE risk (adjusted rate ratio [aRR] 1.42, 1.63, and 2.92 for < or =30 microgram, 31-49 microgram, and 50 microgram, respectively) (22). They also reported that the rate was particularly increased among females who recently started taking oral contraceptive pills (aRR 2.52 [95%CI: 1.14-5.57]) compared with longer-term current users. Estrogen prevents B cell receptor-mediated apoptosis and upregulates several genes that contribute to B cell activation and survival (cd22, shp-1, bcl-2, and vcam-1) (103).

Chemical and physical exposures have also been historically linked to SLE onset, including crystalline silica dust (25, 33, 104, 105), heavy metals such as mercury (43), air pollution and other respiratory particulates (38, 106), residential proximity to hazardous waste sites (26), agricultural pesticides (27, 43, 107), and organic solvents (42, 44). Proposed mechanisms of pathogenesis include stimulation of cellular necrosis and release of intracellular antigens resulting in systemic inflammation and IFN upregulation. These environmental exposures have also been described as important risk factors in the development of RA (42), SSc (44), and vasculitis (48). A comprehensive review of the literature (~1980-2010) on environmental factors and SARD development concluded that among these chemical factors, crystalline silica exposure, solvent exposure, and smoking had the strongest level of evidence (108). Since then, however, multiple studies have been published. The evidence for metal exposure and SARD development including mercury at that time was felt to be insufficient, although there is renewed interest in mercury-induced autoimmunity in more recent studies (109, 110). Mercury exposure has been associated with autoimmune features that are more consistent with pre- or sub-clinical autoimmunity in humans, and in animal studies, acts independently of type I IFN to induce milder disease (111).

UVB radiation can exacerbate pre-existing SLE, however, whether it contributes to SLE disease onset or pathogenesis is less clear. While UVB radiation can up-regulate Th2 cells and down-regulate Th1 cells, induce IL-10 production, increase type I IFN expression, and prolong T cell activation to increase SLE risk (29–31), another subset of UV radiation, UVA, is used as a phototherapy modality to treat cutaneous forms of lupus (112). UVB also has an important role in vitamin D3 synthesis in the skin, which has been hypothesized to *lower* SLE risk (28, 113). Vitamin D deficiency is reportedly common among SLE patients (34) and is important in the regulatory pathways of numerous genes involved in inflammation and immunity including IL-2 inhibition, antibody production, and lymphocyte proliferation (114, 115). We will later discuss a large, randomized, double-blind, placebo-controlled clinical trial called the vitamin D and omega 3 trial (VITAL) trial, where vitamin D 2000 IU daily supplementation was associated with a 22% reduction in the development of autoimmune disease (HR 0.78 [95% CI: 0.61, 0.99], p=0.05) (56).

Viral triggers, particularly Epstein-Barr Virus (EBV), have also been associated with SLE development (35). In a recent study of 436 unaffected SLE patient relatives who were followed for 6.3 ± 3.9 years and evaluated for interim transitioning to SLE, increased serological reactivation of EBV was associated with higher risk of transitioning to SLE (viral capsid antigen IgG OR 1.28 [95%CI: 1.07-1.53], p=0.007 and expression of EBV early antigen IgG (OR 1.43 [95%CI: 1.06-1.93], p=0.02) (36). Proposed mechanisms include molecular mimicry and the release of EBV-encoded small RNAs from infected cells resulting in the induction of type-1 IFN and proinflammatory cytokines via activating toll-like receptor (TLR)-3 signaling (116). The interest in triggering of autoimmune conditions by viral infections was renewed during the coronavirus disease 2019 (COVID-19) pandemic when there were outbreaks of pediatric inflammatory multisystemic syndrome [PIMS also referred to as multisystem inflammatory syndrome in children (MIS-C)] that reportedly followed severe acute respiratory syndrome coronavirus 2 (SARS-CoV-2) infection in children. These reports included cases of Kawasaki-like disease, Kawasaki disease shock syndrome, toxic shock syndrome, myocarditis and macrophage activation syndrome (117–119). In adults, SARS-CoV-2 infection has also been linked to a higher risk of developing a diverse spectrum of new-onset autoimmune diseases as highlighted by two large retrospective studies (50, 120). Chang et al. used data from the TriNetX network and propensity score matching (two cohorts [COVID-19 and non-COVID-19] of 887,455 SARS-CoV-2 unvaccinated individuals) to identify the incidence of autoimmune conditions during the study period (1 January 2020 to 31 December 2021) (50). Unlike EBV, there was a wider spectrum of SARD seen including higher risk of RA (adjusted hazard ratio (aHR) 2.98 [95%CI: 2.78–3.20]), SLE (aHR 2.99 [95%CI: 2.68–3.34]), dermato/polymyositis (aHR 1.96 [95%CI: 1.47–2.61]), SSc (aHR 2.58 [95%CI: 2.02–3.28]), SjD (aHR 2.62 [95%CI: 2.29–3.00]), and other autoimmune diseases. Future studies that elucidate how viruses, such as SARS-CoV-2, increase the risk of SARD development may help implement preventive measures and early treatment in individuals who have had these infections to prevent morbidity and mortality.

A key pathway involved in both anti-viral response and the pathogenesis of SLE and other SARD including IIM and SSc is the type I IFN pathway (121). Approximately 50-70% of adult and pediatric SLE patients have an upregulated IFN signature, a cluster of IFN-stimulated genes, that correlates with disease activity and severity (122). A recent study demonstrated that type-1 IFN inhibits the aryl hydrocarbon receptor (AHR) pathway. Suppressed AHR signaling promotes T cell production of CXC ligand 13 (CXCL13), a chemokine that regulates B cell recruitment and lymphoid aggregation in inflamed tissues (123). AHR is important for sensing changes in the cellular milieu provided by the environment, diet, commensal flora, and host metabolism (124). In response to these environmental ligands, AHR has a protective role against inflammation by downregulating pro-inflammatory pathways (124). In the gut, AHR is expressed in epithelial cells and immune cells in the lamina propria to also stabilize the gut epithelial barrier (124). In the central nervous system, AHR is upregulated in astrocytes and microglia in response to ligands that cross the blood-brain barrier (124). Lower AHR expression has been described as a potential mechanism of pathogenesis for several autoimmune conditions including inflammatory bowel disease (125), multiple sclerosis (126), and psoriasis (127). In SLE, deficits in the AHR-driven immunoregulation exacerbated by the type-1 IFN may explain how alterations in the environment lead to the development of autoimmunity and uncontrolled inflammation. Moreover, polycyclic aromatic hydrocarbons, smoking, air pollution, and other environmental exposures cause DNA methylation changes in the AHR repressor genes, potentially linking these exposures to the development of autoimmunity (128–130). Future studies are warranted to elucidate the pathways by which regulation of the AHR pathway is related to lymphocyte activation status in the pathogenesis of autoimmunity.

### Epigenetic changes

The currently accepted etiologic model for SARD implicates an interaction of inherited genetic factors and environmental exposures over time. DNA methylation (DNAm), an epigenetic change controlling gene expression, is influenced by both genetics and environmental exposures and therefore, may provide a critical link between them [reviewed in (131–133)]. For instance, UV light exposure, infections, silica, heavy metals and pesticide exposures, cigarette smoking, and air pollution are all thought to inhibit DNAm by oxidative stress, which could promote SARD onset specifically or non-specifically (134). In addition to DNAm, cigarette smoking is linked to the activation of enzymes that regulate other types of epigenetic modifications (i.e., post-translational modifications of histones via methylation, acetylation, phosphorylation, ubiquitination, and regulation of non-coding RNA sequences) to mediate the expression of multiple inflammatory genes, thereby participating in the onset development of autoinflammatory diseases (135).

DNAm occurs when a methyl group is added to a cytosine base in a cytosine-phosphate-guanine dinucleotide (CpG) which, in general, silences nearby gene expression. By comparison, demethylation activates gene expression. These changes, mainly demethylation and in particular IFN gene hypomethylation, have been observed in various cell subsets, including CD4 T cells in patients affected by SLE (136–145). Upregulation of type I IFN in SLE is thought to induce an “IFN epigenomic signature”, activating latent enhancers and “bookmarking” chromatin, reprogramming genes to be hyper-responsive, amplifying the inflammatory cascade (146–148). Emerging data reveal that some of these epigenetic changes are correlated with SLE disease manifestations (malar and discoid rash, dsDNA autoantibodies, lupus nephritis) and disease severity (137, 139, 144, 149), and are highly specific to SLE such that they distinguish individuals with existing SLE from controls and other SARD (141, 150). Well-designed epidemiologic studies are still needed to determine whether other epigenetic changes precede the development of SARD and whether such changes could be modified to abrogate disease.

### Microbiome influences

There is mounting evidence that imbalances in the microbiota contribute to metabolic and immune regulatory dysfunction, which may contribute to the pathogenesis of chronic inflammatory diseases such as SARD [reviewed in (151)]. Several independent reported studies of 16S rRNA libraries have identified characteristic patterns of gut dysbiosis in SLE, in which there is an inverse relationship between disease activity and overall biodiversity of the intestinal microbiota (152–154). In studies of 61 female SLE patients, there was an eight-fold increase in *Ruminococcus gnavus* abundance compared to the healthy subjects, and most patients with high R. *gnavus* abundance had active nephritis (152). Increases in *R*. *gnavus* abundance have also been observed in other diseases including allergies and spondyloarthropathies with inflammatory bowel disease (155–157). Importantly, many strains of *R*. *gnavus* express a VH3 B cell repertoire (BCR) targeted B cell superantigen, particularly relevant to SLE given the importance of B cell activation in disease pathogenesis (158).

Evidence suggests that SLE patients may suffer chronic microbial translocation through impaired gut barrier integrity contributing to immunologic dysregulation (159). Oral microbiome studies confirm that SLE patients have a distinct microbiome signature compared to healthy controls, with evidence of translocation of bacteria, e.g., *Veillonella* species, from the oral cavity to the intestine (160, 161).

In healthy adults, the microbiome, even at the level of strains, is relatively stable over many years (162). However, the microbiome can be altered by diet, sleep, exercise, stress, medications (antibiotics and non-antibiotics), and the environment (163). Perturbations in the gut microbiome composition have been suggested to trigger SLE onset or disease flares and *vice versa* (164). In-depth studies examining the impact of lifestyle and environmental factors on changes to the microbiome and subsequent risk of autoimmune diseases are needed.

Other host barriers should also be considered as potential targets for prevention including the oral cavity and lung mucosa as these have been identified as sites of pathogenic autoreactive immune responses that contribute to autoimmune disease. The initiation of RA by inflammation characterized by an aberrant Th-17-dominated immune response, neutrophil activation, antigen citrullination, and anti-cyclic citrullinated peptide (CCP) production is exacerbated by microbial dysbiosis, the presence of oral pathobionts (e.g., *Porphyromonas gingivalis* and *Aggregatibacter actinomycetemcomitans*), and periodontitis has been described (45, 165–167).

The lung mucosa is another site of protein citrullination leading to RA development, promoted by microbial infection or dysbiosis and the inhalation of pollutants such as tobacco smoke or other pollutants (168, 169). This anti-CCP production and translocation into the systemic circulation has been proposed to accelerate the development of RA with interstitial lung disease for individuals who are genetically predisposed (e.g., gain-of-function MUC5B promoter variant reducing mucociliary function in small airways responsible for clearing inhaled particles in the lungs (170)). It is difficult to be certain that microbiome alterations observed in recent studies of SARD patients are not due to established and treated disease. Additional studies of the microbiome before disease onset are warranted.

## Mitigation of environmental factors

### Traditional cohort studies

Our current understanding of lifestyle factors and autoimmune diseases has largely depended on large observational epidemiological studies (53, 54, 171). Many of these studies used self-reported data including the use of validated and standardized questionnaires. These studies also relied on the retention of subjects in the long term to enable repeated measurement of lifestyle behaviors. Nevertheless, these studies have filled important knowledge gaps in our understanding of the link between environmental exposures and autoimmunity.

In the NHS and NHSII cohorts, our group demonstrated that adherence to multiple healthy behaviors (healthy diet (highest 40th percentile of the Alternative Healthy Eating Index), regular exercise (performing at least 19 metabolic equivalent hours of exercise per week), never or past smoker, moderate alcohol consumption (drinking ≥5 gm/day alcohol), and maintaining a healthy body weight (body mass index <25 kg/m2) was associated with a 19% reduction in SLE risk per additional healthy behavior, such that women with four or more healthy lifestyle factors had the lowest risk (HR 0.42 [95%CI: 0.25-0.70]) (53). An even greater reduction per healthy behavior (22%) was observed for the risk of anti-dsDNA-positive SLE. Overall, the population-attributable risk, or the proportion of the risk in this population that could be attributed to these five modifiable lifestyle risk factors was 47.7% [95%CI: 23.1-66.6%]. Using the same cohorts and similar modeling, a lower risk of RA was also observed with a healthier lifestyle, i.e., women with five healthy lifestyle factors had the lowest risk (HR 0.42 [95%CI: 0.22-0.80]) (54). Therefore, a significant proportion of the risks of both SLE and RA may be preventable by adhering to healthy lifestyles.

### Intervention and prevention trials

There is a scarcity of clinical trials examining lifestyle and environmental interventions and prevention strategies to reduce the risk of autoimmune disease development. One of the challenges in designing a strong and well-powered prevention study is identifying which at-risk individuals to study. Our group has previously developed SLE risk prediction models having 76% accuracy by combining family history, genetic factors, and lifestyle, medical and behavioral exposures that classify a woman’s risk of SLE in the next two years (172). There is also a rapidly growing panel of potential biomarkers of SLE risk or early disease including anti-dense fine speckled 70 (DFS70) as a rule-out SARD test (173), anti-C1q antibodies as a rule-in test (174), cytokines and chemokines (175, 176), IFN signature (177), as well as markers of complement activation (178). Therefore, identifying individuals for screening, risk-stratifying, assessing biomarkers, and testing intervention and prevention strategies before clinical disease onset has recently become possible (65, 179).

In a pivotal randomized, double-blind, placebo-controlled vitamin D and omega 3 trial (VITAL) trial with a two-by-two factorial design (n=25 871 participants followed for a median of 5.3 years), vitamin D (2000IU/day) supplementation for five years [with or without omega 3 fatty acid (1000 mg/day)] had a significant reduction in the risk of confirmed autoimmune disease of 22% (HR 0.78 [95% CI: 0.61, 0.99], p=0.05) (56). This included RA, polymyalgia rheumatica, autoimmune thyroid disease, psoriasis, inflammatory bowel disease, and others (e.g., SLE, SSc). Individuals who received an omega-3 fatty acid supplementation (with or without vitamin D supplementation) had a reduced rate of incident autoimmune disease by 15% but this was not statistically significant. However, the two-year post-intervention observation study where participants were no longer provided with any supplements but were invited to continue being observed while off assigned supplements, demonstrated that the protective effects of the 5.3 years of randomized exposure to 2000 IU/day of vitamin D dissipated, but the randomized supplementation with 1,000 mg/day of omega-3 fatty acids for the 5.3 years was seen to have a sustained effect in reducing autoimmune disease incidence (180). The results suggest that vitamin D supplementation of 2000 IU/day should be given continuously for long-term prevention of autoimmune disease, while the beneficial effects of omega-3 fatty acids may be more sustained.

The only SLE-specific prevention trial to date is the “Study of Anti-Malarials in Incomplete Lupus Erythematosus (SMILE)” (61), which was set to determine whether SLE progression can be abrogated by using hydroxychloroquine (HCQ) among patients with a positive ANA test and at least one (but not three or more) additional clinical or laboratory criterion from the 2012 Systemic Lupus Inception Collaborating Clinics (SLICC) classification criteria (181). This highly anticipated, multicenter, randomized, double-blind, placebo-controlled, 24-month trial is expected to be completed soon.

A similar HCQ prevention trial in RA (“Strategy to Prevent the Onset of Clinically-Apparent Rheumatoid Arthritis” or STOP-RA) was halted early due to the futility of the treatment (182). In the interim analysis it was observed that in individuals who were anti-CCP positive but without inflammatory arthritis at baseline, one year of HCQ was not superior to placebo in preventing or delaying the development of inflammatory arthritis, and the classification of individuals as having RA at 3 years (probabilities of RA development were 34% in the HCQ arm and 36% in the placebo; p=0.844). Therefore, in RA, HCQ did not help prevent or delay the onset of clinical disease compared to placebo. The study did suggest however that anti-CCP at levels of ≥40 units will be an important enrolment criterion in future RA prevention studies. Therefore, as we strive towards a future of prevention over cure in any SARD, a better and more standardized approach to identifying the timing of intervention and which patients are at the highest risk is urgently needed to ensure the success of prevention trials.

Other RA prevention trials such as the “TREAT Early Arthralgia to Reverse or Limit Impending Exacerbation to Rheumatoid arthritis” (TREAT EARLIER) trial examining one year of methotrexate also did not meet its endpoint of development of clinical arthritis among individuals with arthralgia clinically suspected of progressing to RA and magnetic resonance imaging (MRI)-detected subclinical joint inflammation (183). The T-cell co-stimulation inhibitor abatacept has shown greater promise in delaying RA development in two different at-risk populations. In the “Abatacept inhibits inflammation and onset of rheumatoid arthritis in individuals at high risk” or ARIAA trial, abatacept treatment for six months among RA-at-risk individuals (anti-CCP positive and showing MRI signs of inflammation) reduced subclinical joint inflammation and delays the development of RA (62). In the “Arthritis Prevention In the Pre-clinical Phase of RA with Abatacept” (APIPPRA) trial, at-risk individuals were defined as individuals with arthralgia, anti-CCP plus rheumatoid factor (RF) positive or high anti-CCP titers ≥3 x upper limit of normal plus RF negative, without synovitis at baseline (63). In this randomized, double-blind, multicenter, parallel, placebo-controlled, phase 2b clinical trial, 52 weeks of abatacept treatment reduced RA development over two years compared to placebo. However, by 24 months, the effect of abatacept treatment on symptom burden and subclinical inflammation as determined by ultrasound was not sustained. Therefore, longer treatment with abatacept beyond 12 months might be required. These studies again highlight the need for criteria that identify at-risk individuals from patients with early RA and the most appropriate time to target preventative interventions (184).

### Future technologies for research on environmental exposures and SARD

In the last decade, there has been an exponential uptake of AI technologies to study diseases including SARD [reviewed in (185–187)]. Much of this is due to greater access to a variety of data sources, e.g., images, efficient data collection tools, and supercomputer and analytic methods to rapidly compute. ML is a type of AI that refers to utilizing computers to perform specific tasks by learning from the data rather than being explicitly programmed with instructions such as traditional statistical tests. Within ML, different algorithms are generally categorized into supervised, unsupervised, reinforcement, and deep learning.

In the study of SARD, ML has proven useful in developing prediction models for diagnosis and disease outcomes and in elucidating pathogenesis [reviewed in (185)]. As SARD are highly complex, multifactorial, and heterogeneous diseases, ML is an ideal approach because it can reveal patterns and interactions between variables in large and complex datasets more accurately and efficiently than traditional statistical methods. As we enter an era of ‘multi-omics’, information on our patients is becoming increasingly ‘layered’ and challenging to interpret and ML holds promise for new insights and interpretations.

Utilizing ML, we recently demonstrated that there are four unique SLE clusters defined by longitudinal autoantibody profiles alone (188). While these clusters are predictive of disease activity, treatment requirements, complications, and mortality, it also points to autoantibodies as being a fundamental underlying mechanism of immune dysregulation and disease pathogenesis of SLE. This approach can be adopted to study pathogenesis for other SARD and inform more personalized monitoring and treatment plans. The focus of current SLE ML models is on the identification of patients with established disease or the prediction of specific SLE manifestations, e.g., nephritis, neuropsychiatric disease. This includes a validated diagnostic algorithm called the SLE Risk Probability Index (SLERPI) where a SLERPI score of greater than 7 was highly accurate (94.2%) and sensitive for detecting early disease (93.8%) and severe manifestations including kidney (97.9%) and neuropsychiatric involvement (91.8%) (189). Future studies to develop ML models that predict the development of new-onset SLE utilizing datasets that include environmental exposures are needed.

## Conclusions

Our examination of risk and protective factors for SARD development, including adherence to multiple healthy lifestyle behaviors, has helped our understanding of the pathogenesis of autoimmunity that involves immune dysregulation, epigenetics, and an altered microbiome. Multiple environmental exposures, including social and behavioral factors throughout our lifespan are likely synergistic and interactive with each other and with genetic factors, influencing the immune system in a complex interplay of epigenetic, hormonal, and microbiome influences, leading to systemic inflammation and eventual organ damage in some. While a major focus has been placed on identifying new targets for disease treatment, shifting the care paradigm to disease prevention is an attractive proposition, especially as our ability to identify high-risk individuals improves. In the few prevention trials that have been conducted, the importance of identifying patients at the highest risk and the likelihood of benefiting from preventative treatment has been highlighted, and thus far, biomarkers have played a critical role in risk stratification. Given the complexity and vast clinical heterogeneity of SARD, ML approaches will become increasingly relied upon to study SARD pathogenesis and prevention.
